# Data on the relationship between caffeine addiction and stress among Lebanese medical students in Lebanon

**DOI:** 10.1016/j.dib.2019.104845

**Published:** 2019-11-28

**Authors:** Ali Samaha, Ahmad Al Tassi, Najwa Yahfoufi, Maya Gebbawi, Mohammad Rached, Mirna A. Fawaz

**Affiliations:** a Department of Biomedical Sciences, Lebanese International University, Lebanon; b Beirut Arab University, Faculty of Health Sciences, Beirut, Lebanon; c Lebanese University, Faculty of Public Health IV, Zahle, Lebanon; d Lebanese University, Doctoral School for Literature and Social Sciences, Bierut, Lebanon

**Keywords:** Stress disorders, Behaviour, Addictive, Caffeine

## Abstract

Stress continues to be a global burden. It may be thought of as necessary to human thriving; however, challenging and unfavorable functioning may take place when many significant stressors are imposed repetitively or concurrently without resolve. Research suggests that medical students perceive higher levels of stress than students in other health-related disciplines [1–3]. Since caffeine is a psychoactive substance that stimulates the central nervous system, medical students use to consume it more than other students to overcome the stress they face due to studying. The paucity of knowledge regarding the trends of caffeine consumption among medical students in developed countries and especially in Lebanon has encouraged us to examine the relationship between caffeine addiction and stress among Lebanese medical students in Lebanon. A non-experimental cross-sectional correlational design was employed to gather data from a sample of 800 medical students enrolled in different studying years in different Lebanese universities. Well-established psychometric instruments were used in primary data collection method, which are the Medical Student Stressor Questionnaire (MSSQ) and the Caffeine Consumption and Dependence Scale. The analyzed data is provided in the tables included in this article.

**Table:** Specifications Table

Subject area	*Psychology*
More specific subject area	*Stress and Addiction*
Type of data	*Tables*
How data was acquired	*Quantitative Questionnaires: MSSQ and The Caffeine consumption and dependence Scale*
Data format	*Raw and Analyzed*
Experimental factors	- *Convenience Sample consisted of 800 medical students from various academic years.* - *Informed consent was obtained and signed.* - *Participation was anonymous and voluntary.* - *Comparison between different universities was omitted as requested by the majority of institutions' authorities.*
Experimental features	*The researchers measured the stress among medical students using the Medical Student Stressor Questionnaire (MSSQ) and the caffeine addiction using the Caffeine Consumption and Dependence Scale.*
Data source location	*Lebanon*
Data accessibility	*Data is available within this article*
Related research article	*Ríos, J. L., Betancourt, J., Pagan, I., Fabián, C., Cruz, S. Y., González, A. M., ... & Palacios, C. (2013). Caffeinated-beverage consumption and its association with socio-demographic characteristics and self perceived academic stress in first and second year students at the University of Puerto Rico Medical Sciences Campus (UPR-MSC). Puerto Rico health sciences journal, 32(2).*

**Table:** 

**Value of the Data** • The data provided in this paper may be used to increase awareness about the overlooked issue of caffeine addiction and stress among medical students. • The data showed a high impact of academic issues such as academic year, having stressful social events, low socio-economic status, and being forced to study medicine on medical students stress and performance. This mandates thorough actions to be considered by both medical institutions and medical students to fight this stress and maintain a healthier life and academic development. • Our data are concurrent with previous research studies, thus making it of interest to other researchers. This topic was tackled by multiple previous studies in Lebanon, where it found that Lebanese medical students and mainly 97% of them were unconsciously becoming addicted to caffeinated substances [[1], [2], [3], [4]], and showing signs of anxiety, burnout and depression [[5], [6], [7], [8], [9]]. • The data can be used to carry out comparative studies in the same field of research.

## 1 Data

The data shows that the mean age of the participants is 21.92 ± 2.16 years, and that the majority of respondents belong to the first four academic years (462 students). Table 1, Table 2 provide more details regarding the demographic characteristics of medical students who participated, while Table 3 shows the perceived stressors measured by the MSSQ. The data indicate that 396 students (66.44%) are subject to high and severe academic related stress and that 31.2% report equally high and severe stress related to teaching and learning and social related stressors domains. Group activity is reported high and severely stressful by 180 students (30.2%). The inter and intrapersonal related stressor (IRS) and the drive and desire related stressor (DRS) seem to have minor effects of students; respectively 168 (28.18%) and 90 (15.1%) students report them as causing high and severe stress. Taking into consideration the general average of each stress domain as calculated using MSSQ, few significant data were noted. However, the data shows that there is a significant difference related to gender between ARS, IRS and TLRS with females are more prone to be stressful in the three domains as reported respectively by general means (2.815, 1.679 and 1.852). Another significant difference is noted between income groups and ARS and IRS; in both domains students belonging to high-income families are less subjected to stress in both domains with respective general means of 2.161 and 1.021. Nevertheless no significant differences are noted between different stress domains, parents' status and other demographics (omitted from analysis) (Table 4, Table 5). Moreover, the value of Pearson's correlation coefficient has been calculated among different stress domains and positive correlation was found among all domains. All correlations were significant at the 0.01 level (Table 6). The highest correlation is found between academic related stressors (ARS) and group activity related stressors (GARS), the latter correlates strongly with the teaching and learning related stressors domain. The intra and inter-personal stressors' domain correlates positively and significantly with social related stressors (SRS), correlating largely at its turn with both TLRS and IRS. Although small correlation is noted between DRS (drive and desire related stressors) and ARS, a strong one prevails between the former and TLRS. Table 7, Table 8, Table 9 show the descriptive data regarding caffeine consumption pattern, sources and addiction. Table 10 represents the Caffeine toxicity and withdrawal symptoms which were evaluated among participants reporting regular caffeine intake (446 students). Restlessness, nervousness and anxiety are the most reported symptoms. In addition, Table 11 highlights the main encountered withdrawal symptoms and their relative occurrence rates among participants. A Pearson's correlation test was carried out to examine the relationship between caffeine intake and various relevant variables. The data showed a significant correlation between daily caffeine intake, caffeine intake in Kg of body weight and random plasma caffeine level with Pearson's coefficients of 0.955 and 0.747 respectively. Also, a significant correlation was found among the daily time spent online and the daily caffeine intake and caffeinemia (0.988 and 0.985 respectively), smoking occupies the second place correlating largely with caffeine intake (0.971) and plasma caffeine (0.573) as shown in Table 12. Furthermore, another Pearson's correlation test was carried out to examine the relationship between caffeine intake and caffeinemia on one hand and the stress domains on the other hand. The data showed that daily caffeine intake was significantly correlated with IRS (0.138), DRS (0.272) and TLRD (0.161), while caffeinemia was also strongly correlated with IRS (0.405), DRS (0.407) and TLRD (0.195) (Table 9). The questionnaires used to obtain the data are provided as a supplementary file to this article.

**Table 1:** MSSQ perceived stressors.

Level of stress	ARS		IRS		TLRS		SRS		DRS		GRAS	
	N	%	N	%	N	%	N	%	N	%	N	%
None	24	4	24	4	22	3.7	28	4.7	14	2.3	26	4.4
Mild	46	7.7	232	39.3	204	34.2	158	26.5	368	61.7	188	31.5
Moderate	130	21.8	172	28.9	184	30.9	224	37.6	124	20.8	202	33.9
High	206	34.6	124	20.8	142	23.8	128	21.5	48	8.1	134	22.5
severe	190	31.9	44	7	44	7.4	58	9.7	42	7	46	7.7
Total	596		596		596		596		596		596

**Table 2:** Stress domains and gender.

Stress domain	Gender	Mean ± Standard deviation	P value
ARS	Male	2.234 ± 1.038	<0.001
	Female	2.815 ± 0.838	
IRS	Male	1.233 ± 1.041	0.005
	Female	1.619 ± 1.103	
TLRS	Male	1.441 ± 1.033	0.003
	Female	1.825 ± 1.028	
SRS	Male	1.572 ± 0.959	0.19
	Female	1.876 ± 1.032	
DRS	Male	0.856 ± 1.052	0.26
	Female	1.571 ± 1.005	
GARS	Male	1.571 ± 1.005	0.8
	Female	1.808 ± 0.961

**Table 3:** Caffeine consumption pattern and caffeinemia.

Caffeine concentration	Mean	Standard deviation
Daily caffeine intake in milligrams/day	193.32	361.81
Daily Caffeine intake in milligrams per KG of body weight per day	2.807	5.17
Random Plasma caffeine level in microgram/ml	16.495	12.32

**Table 4:** Main reported sources of caffeine.

Source of caffeine	N	%
Coffee and its derivatives	528	88.59
Coca and its derivatives	368	61.75
Energy drinks	209	35.06
Tea	170	28.52
Artificial juices	138	23.15

**Table 5:** Caffeine addiction survey.

Item	Description	Number of Yes responses	%
1	I believe caffeine enhances performance (athletic, academic, artistic, etc).	402	67.45
2	I believe that caffeine can be harmful to my health and can hurt me.	408	68.45
3	I believe caffeine is addictive.	430	72.15
4	I believe that caffeine can disrupt coordination.	308	51.67
5	I have religious objections to caffeine consumption.	120	20.13
6	Have you ever used caffeine to wake up in the morning?	344	57.72
7	Have you ever used caffeine to stay awake?	416	69.8
8	Have you ever used caffeine to enhance physical performance?	266	44.63
9	Have you ever used caffeine to enhance mental performance?	366	61.4
10	Have you ever used drinks/pills with caffeine to lose weight?	96	16.1
11	Do you drink caffeine containing beverages on a daily basis (e.g. coffee, tea, soft drinks, etc)?	446	74.83

**Table 6:** Undesirable caffeine effects/caffeine toxicity.

**Sleep and anxiety related**		
Inability to sleep	34	7.62%
Inability to concentrate	30	6.72%
Restlessness	240	53.81%
Excitement	34	7.62%
Irritation	38	8.52%
Hyperactivity	50	11.21%
Nervousness	210	47.08%
Anxiety	200	44.84%
**Somatic related**		
Red face	32	7.17%
Hot flashes	22	4.93%
Hives	70	15.69%
Stomach aches	54	12.1%
Headaches	28	6.27%
Muscular twitches	18	4.03%
Fast heart beats	144	32.28%
Irregular heart beats	178	39.91%
Rambling speech	68	15.24%

**Table 7:** Caffeine withdrawal symptoms.

Withdrawal symptoms	N	%
Fatigue	174	39.01
Drowsiness	94	21.07
Depression and or anxiety	70	15.96
Stomach aches	34	7.62
Vomiting	24	5.38
Headaches	148	33.18
Irritability	108	24.21
Craving for caffeine	142	31.83

**Table 8:** Pearsons' correlation coefficient between caffeine related variables and some demographics.

	Daily caffeine intake	Plasma caffeine level
Living conditions	0.078	0.09
Daily time spent on internet	0.988	0.985
Smoking and Hubble bubble	0.971	0.573
Facebook account	0.365	0.688
Rate of application use	0.921	0.438
Adult sites visits	0.783	0.569

**Table 9:** Pearsons' correlation coefficient between different stress domains, GPA categories, IAT categories, daily Caffeine intake and plasma caffeine level.

	ARS	IRS	TLRS	SRS	DRS	GARS
Daily Caffeine intake	−0.15	0.138^a^	0.161^a^	0.106	0.272^b^	0.041
Caffeinemia	−0.056	0.405^b^	0.195^a^	0.047	0.407^b^	−0.028

a Significant data at 0.05.

b Significant data at 0.01.

**Table 10:** Undesirable caffeine effects/caffeine toxicity.

**Sleep and anxiety related**		
Inability to sleep	34	7.62%
Inability to concentrate	30	6.72%
Restlessness	240	53.81%
Excitement	34	7.62%
Irritation	38	8.52%
Hyperactivity	50	11.21%
Nervousness	210	47.08%
Anxiety	200	44.84%
**Somatic related**		
Red face	32	7.17%
Hot flashes	22	4.93%
Hives	70	15.69%
Stomach aches	54	12.1%
Headaches	28	6.27%
Muscular twitches	18	4.03%
Fast heart beats	144	32.28%
Irregular heart beats	178	39.91%
Rambling speech	68	15.24%

**Table 11:** Caffeine withdrawal symptoms.

Withdrawal symptoms	N	%
Fatigue	174	39.01
Drowsiness	94	21.07
Depression and or anxiety	70	15.96
Stomach aches	34	7.62
Vomiting	24	5.38
Headaches	148	33.18
Irritability	108	24.21
Craving for caffeine	142	31.83

**Table 12:** Pearsons' correlation coefficient between caffeine related variables and some demographics.

	Daily caffeine intake	Plasma caffeine level
Living conditions	0.078	0.09
Daily time spent on internet	0.988	0.985
Smoking and Hubble bubble	0.971	0.573
Facebook account	0.365	0.688
Rate of application use	0.921	0.438
Adult sites visits	0.783	0.569

## 2 Experimental design, materials, and methods

### 2.1 Design

A cross sectional descriptive correlational design was used to asses and quantify the main sources of stress, caffeine consumption, caffeine intake behaviors, and examine the relationship between the stress and caffeine.

### 2.2 Sample and settings

A convenience sample of medical students enrolled in different studying years in different Lebanese universities was adopted. A total of 800 students were approached to participate in the data collection, 720 of them consented for enrollment (90% respond rate) and only 596 students have completed appropriately and fully the questionnaire to be suitable for analysis. The ethical approval was obtained from Institutional Review Board of Beirut Arab University. The students were approached by the researcher, where the aim of the study was explained, and participants were informed participation is voluntary and anonymous, then they were asked to sign an informed consent, and then fill the paper-based questionnaires after explaining the items. The students were sampled from medical schools that follow the Lebanese educational system, where students need to finish 6 years of education to graduate as general physicians.

### 2.3 Questionnaires

Well-established psychometric instruments were used in the data collection method. The first questionnaire employed was the short version of the Medical Student Stressor Questionnaire (MSSQ) which consists of 20 items representing the six main stressor domains studied among medical students [10,11]. Stressors are grouped in six hypothetical groups: academic related stressors (ARS), intrapersonal and interpersonal related stressors (IRS), teaching and learning-related stressors (TLRS), social related stressors (SRS), drive and desire related stressors (DRS), and group activities related stressors (GARS). Based on score analysis perceived stress in each category is classified as mild, moderate, high and severe with respective scores of 0.00–1.00, 1.01–2.00, 2.01–3.00 and 3.01–4.00. The validation found that the MSSQ has good psychometric properties; it is a valid and reliable instrument that can be used to identify students' stressors as well as measure the intensity of the stressors. Factor analysis shows that all the items are well distributed according to the six groups. Reliability analysis shows that the MSSQ has a high internal consistency as Cronbach's alpha coefficient value was 0.95. The Caffeine consumption and dependence Scale: The Substance Abuse Module (SAM) was the other questionnaire used for the data and was the only available structured interview that assesses caffeine dependence based on the International Diagnostic Interview-Substance Abuse Module (DSM V) criteria. This scale consisted of 7 questions, their answers yield a diagnostic algorithm that was developed by the Washington University team and checked by members of the DSM-IV Field Trials. Daily caffeine consumption was calculated based on the daily intake of its different sources: coffee and its derivatives, soft drinks and energetic drinks. Beside a random plasma caffeine levels using high performance liquid chromatography was measured after blood collection from willing and consenting participants [12].

### 2.4 Statistical analysis

Data entry and analysis were performed using Statistical Package for the Social Sciences (SPSS) Version 24 [13]. Descriptive data are reported as means and standard deviations or as percentages. Correlational analyses were used to assess relationships between studied variables.
