# Supplementary material for: Data on the relationship between caffeine addiction and stress among Lebanese medical students in Lebanon
Source: Data Brief. 2019 Nov 28;28:104845. doi: 10.1016/j.dib.2019.104845 (PMC6909133; doi:10.1016/j.dib.2019.104845)
Supplement: Multimedia component 2 [file mmc2.docx]

**The Medical Student Stressor Questionnaire (MSSQ)**

1- Tests/examinations

2 -Falling behind in reading schedule

3- Large amount of content to be learnt

4- Lack of time to review what have been learnt

5- Heavy workload

6- Verbal or physical abuse by other student(s)

7- Verbal or physical abuse by teacher(s)

8-Verbal or physical abuse by personnel(s)

9- Conflict with teacher(s)

10- Not enough feedback from teacher (s)

11- Uncertainty of what is expected of me

12- Lack of recognition for work done

13- Unable to answer questions from patients

14- Talking to patients about personal problems

15- Facing illness or death of the patients

16- Unwillingness to study medicine

17- Parental wish for you to study medicine

18- Participation in class presentation

19- Need to do well (imposed by others)

20-Feeling of incompetence

**Yusoff, M. S. B. (2011). The validity and reliability of secondary school stressor questionnaire (3SQ) in identifying stressor among adolescents in secondary school. Int Med J, 18(2), 99e105.‏**

**The Caffeine consumption and dependence Scale**

| **Item** | **Description** | **Yes** | **No** |
| --- | --- | --- | --- |
| 1 | I believe caffeine enhances performance (athletic, academic, artistic, etc). |  |  |
| 2 | I believe that caffeine can be harmful to my health and can hurt me. |  |  |
| 3 | I believe caffeine is addictive. |  |  |
| 4 | I believe that caffeine can disrupt coordination. |  |  |
| 5 | I have religious objections to caffeine consumption. |  |  |
| 6 | Have you ever used caffeine to wake up in the morning? |  |  |
| 7 | Have you ever used caffeine to stay awake? |  |  |
| 8 | Have you ever used caffeine to enhance physical performance? |  |  |
| 9 | Have you ever used caffeine to enhance mental performance? |  |  |
| 10 | Have you ever used drinks/pills with caffeine to lose weight? |  |  |
| 11 | Do you drink caffeine containing beverages on a daily basis (e.g. coffee, tea, soft drinks, etc)? |  |  |

**Foa, E. B., & Kozak, M. J. (1995). DSM-IV field trial: obsessive-compulsive disorder. The American journal of psychiatry.‏**
