# Supplementary material for: Data on the relationship between caffeine addiction and stress among Lebanese medical students in Lebanon
Source: Data Brief. 2019 Nov 28;28:104845. doi: 10.1016/j.dib.2019.104845 (PMC6909133; doi:10.1016/j.dib.2019.104845)
Supplement: Multimedia component 3 [file mmc3.pdf]

ResearchGate

See discussions, stats, and author profiles for this publication at: https://www.researchgate.net/publication/200640404

The Medical Student Stressor Questionnaire (MSSQ) Manual

Chapter · February 2010

CITATIONS
15

READS
23,580

2 authors, including:

Muhamad Saiful Bahri Yusoff
Universiti Sains Malaysia

276 PUBLICATIONS **1,388** CITATIONS

SEE PROFILE

Some of the authors of this publication are also working on these related projects:

Project Rethinking Education Environment: The Clinical Education Environment Framework View project

Project Research View project

All content following this page was uploaded by Muhamad Saiful Bahri Yusoff on 22 May 2014.

The user has requested enhancement of the downloaded file.

The Medical Student Stressor Questionnaire (MSSQ) Manual

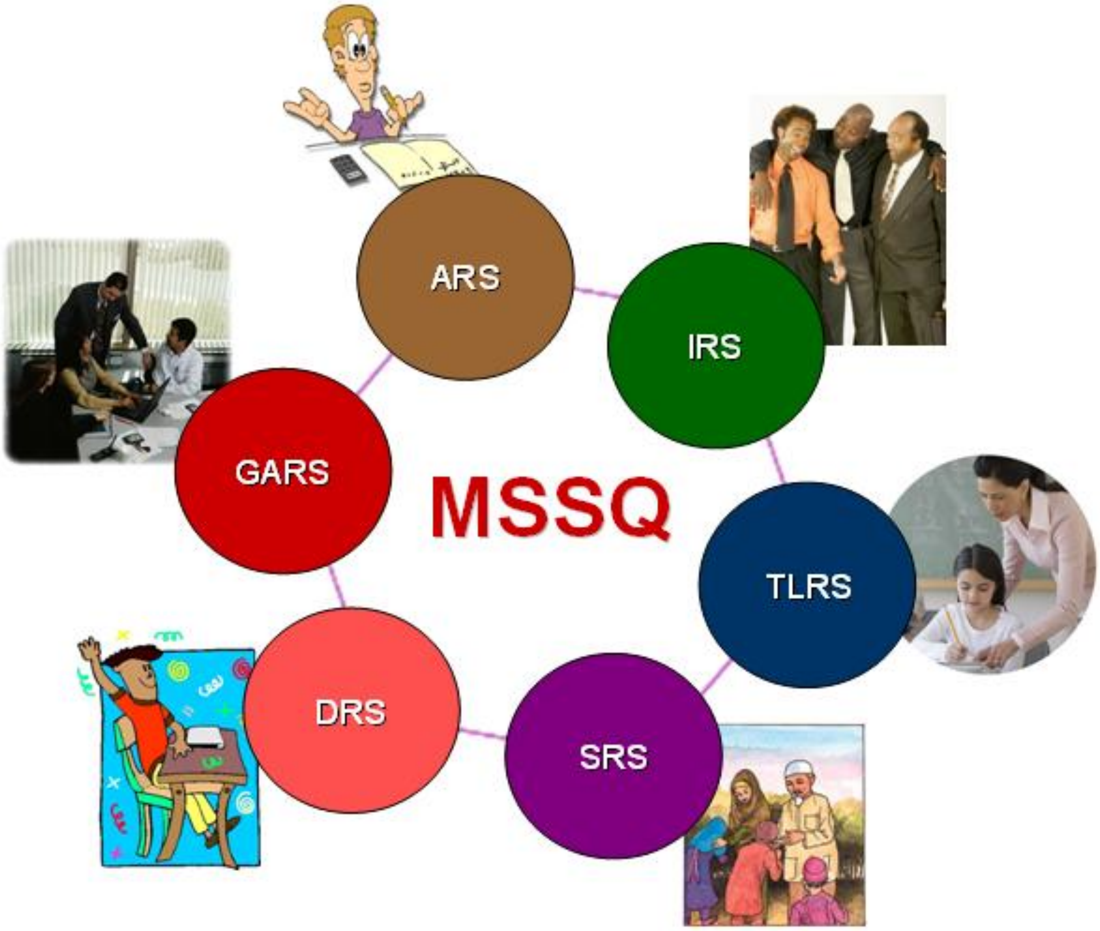

An explanatory guide on stress and stressors in medical study to help you understand what stresses you and how to produce positive outcomes from it.

Muhamad Saiful Bahri Yusoff & Ahmad Fuad Abdul Rahim

First Publication: February 2010

© Dr Muhamad Saiful Bahri Yusoff

© All rights reserved. No part of this publication may be reproduced stored in a retrieval system, or transmitted, in any form or by any means, electronic, mechanical, photocopying, recording, or otherwise, without prior permission of author/s or publisher.

ISBN: 978-967-5547-01-0

Edited by

Dr Muhamad Saiful Bahri Yusoff
&
Dr Ahmad Fuad Abdul rahim

Medical Education Department,
School of Medical Sciences,
USM, Kubang Kerian,
16150 Kota Bharu
Kelantan, Malaysia

Published by:

KKMED Publications
Medical Education Department,
School of Medical Sciences,
USM, Kubang Kerian,
16150 Kota Bharu
Kelantan, Malaysia

Published in Malaysia

Contents

Stress models and its relationship with undergraduate medical training..... 3
Medical students and stress 6
Stressors of medical students 8
The Medical Student Stressor Questionnaire (MSSQ) 9
The validity of the MSSQ... 10
Significance of MSSQ scores 11
Mild, Moderate, High & Severe scores: what do they mean? 12
Detailed Description of the Six Stressor Domains 13
DOMAIN I: Academic Related Stressor (ARS) 13
DOMAIN II: Interpersonal & Intrapersonal Related Stressor (IRS) 14
DOMAIN III: Teaching and Learning Related Stressor (TLRS) 14
DOMAIN IV: Social Related Stressor (SRS).. 15
DOMAIN V: Drive & Desire Related Stressor (DRS) 15
DOMAIN VI: Group Activities Related Stressor (GARS) 16
The Medical Student Stressor Questionnaire (MSSQ) 17
How to score the questionnaire.. 19
Where to get the full version of MSSQ... 21
References 22

Stress models and its relationship with undergraduate medical training

Stress is defined as the body's nonspecific response or reaction to demands made on it, or to disturbing events in the environment (1,2). It is not just a stimulus or a response but it is a process by which we perceive and cope with environmental threats and challenges (3). Personal and environmental events that cause stress are known as stressors (4, 5). In short, stress is emotional disturbances or changes caused by stressors. Linn & Zeppa (6) stated that some stress in medical school training is needed for learning. Stress which promotes and facilitates learning is called 'favourable stress' and stress which inhibits and suppresses learning is called 'unfavourable stress'. The same stressors may be perceived differently by different medical students, depending on their cultural background, personal traits, experience and coping skills.

Numerous stress models have been presented to address the question of work stress and how the individual reacts to this (7). According to the job-strain model, introduced by Karasek & Theorell in 1990, there are two factors that determine stress; decision autonomy and psychological demands (8). Persons who have a sense of control over their work are bothered less by stress. Such persons identify changes and problems as challenges rather than threats. This model predicts that the greatest stress will be in jobs or situations characterized by high psychological demands and low decision autonomy such as in medical study. In this model, stress is viewed as a function of the job rather than the person.

In the person-environment fit model of stress, introduced by Van Harrison in 1978, stress is seen as the result of a mismatch between the requirements and demands of the job and the person's real or perceived ability to meet these demands (9). In this model, stress arises from the conflict people may feel between the different roles expected of them and their personal ability to meet these different tasks. Task conflict can also result in stress, such as in the situation of medical students who are unclear

about expectations by their medical teachers. The students are confronted by demands and expectations from a number of sources that cannot all be met within the time given. Task ambiguity can also result in stress, such as when students are presented with inadequate or unclear information about their tasks. They are uncertain about what they are expected to do. Filley & House (10) reported that such conflicts lead to reduced work satisfaction and has an adverse effect on individual performance.

Siegrist has introduced the effort-reward model in 1996; in this model stress is believed to occur when the amount of effort required and expended exceeds the occupational rewards such as recognition of and feedback on work done (11). Brissei et al. (12) reported that those who are satisfied with their occupational rewards have lower levels of burnout. In the medical training context, there is a concern about the insufficiency of recognition and feedback to medical students compared to the effort that have been given to their work. This is a possible explanation for the high prevalence of distress among medical students as reported by many studies (13 - 18).

Another model of stress is the demands-supports-constraints model introduced by Payne & Fletcher in 1983. This model posits that constraints on a person, in the form of lack of support or resources in the context of high demand lead to stress (19). Brissie et al. (12) found that persons who feel well supported are less likely to burn out. Logically it can be understood because doing extra work with the same or insufficient support (either from family, friends or teachers) and resources is likely to contribute to stress. In the medical training context, there is a concern about the insufficiency and ineffectiveness of the student support system for medical students compared to the overload of work demanded from the students. This is another possible explanation and reason for the high prevalence of distress among medical students as reported by many studies (13-18).

Thus stress in medical training can create a serious problem if it is uncontrolled. Its importance for undergraduate medical students can be summarized in the following equation:

$$UGS = [\{Ac \times (S/T) \times As \times IL\} + TC + TA] - [SA + RLS + RA + SU]$$

UGS	= Undergraduate stress
Ac	= Academic workload (hours of teaching and learning per week)
T	= Number of teachers
S	= Number of students
As	= Assignment workload
IL	= Intensity of learning (new subjects taught to student per week)
TC	= Task Conflict
TA	= Task Ambiguity
SA	= Students' Autonomy
RLS	= Recognition & Learning Satisfaction
RA	= Resources available
SU	= Support Unit (mentally, emotionally, spiritually & physically).

Medical students and stress

Tertiary education has always been regarded as highly stressful environment to students (16, 17). Medical training further adds to the already stressful environment. Studies have revealed a high prevalence of stress in medical students, ranging from 30% to 50% (13-18). Two studies in a Malaysian government university reported that 29.1 % to 41.9% of the medical students surveyed had emotional disorder (13, 17) and another study in a Malaysian private medical school reported that 46.2% had emotional disorders (16) as measured by GHQ-12. Apart from that, the stress level is higher in medical students compared to students in other courses. A study in Singapore reported that 57% of medical students had emotional disorder compared to 47.3% of law students as measured by GHQ (20). Another study in Turkey reported that 47.9% of medical students had emotional disorder compared to 29.2% of economic and physical education students as measured by GHQ (21). The alarming facts suggested a situation of elevated psychological pressure on medical students.

Chronic exposure to stressful conditions exerts negative effects on emotional, mental and physical well-being of students. Numerous studies have revealed that persistent stressful conditions were associated with mental and physical health problems in medical students at various stages of their training (13-18). Studies have reported an association of excessive stress level with lowered medical students' self-esteem (6, 22), anxiety and depression (23, 24), difficulties in solving interpersonal conflicts (25), sleeping disorders (26, 27), increased alcohol and drug consumption (28-30), cynicism, decreased attention, reduced concentration and academic dishonesty (31). It is also associated with inhibition of students' academic achievement and personal growth development (6). Excessive stress was also linked with medical student suicide (36). As a result, medical students may feel inadequate and unsatisfied with their career as a medical practitioner in the future (17). It is noteworthy that many researchers have stated the importance of early diagnosis as well as effective

intervention programme, which can prevent possible future illnesses among medical students (16, 21).

Stressors of medical students

A stressor is defined as a personal or environmental event that causes stress (4, 5). Stressors of medical students generally were grouped into six categories; academic related stressors (ARS), intrapersonal and interpersonal related stressors (IRS), teaching and learning-related stressors (TLRS), social related stressors (SRS), drive and desire related stressors (DRS), and group activities related stressors (GARS) (14). Studies have revealed that the stressors affecting medical students' well-being seem to be related to the medical training, especially academic matters (14, 17, 21, 32-34). They found that the top four stressors were tests and examinations, time pressure, too much content to be studied, and getting behind in work. Another three common stressors were conflicting demands, not getting work done within time planned and heavy workload. A small number of medical students suffer from personal problems, but the effect of this on student psychological morbidity and academic success is unclear (17, 31). Curriculum differences in medical schools may not necessarily cause differences in the overall pattern of stressors (i.e. most of the top stressors are related to academic matters), although frequency (rank) of some stressors may be significantly different (33, 34).

The Medical Student Stressor Questionnaire (MSSQ)

The MSSQ was developed to identify the stressors of medical students as well as measure the intensity of stress caused by the stressors. The six domains of stress measured by the MSSQ were developed based on various researches. The items of MSSQ were selected from literature review related to stress researches. All the items were designed based on its suitability and compatibility with the local cultures and values.

The SSQ grouped stressors into six domains, each based on a common underlying theme:

1. Academic related stressors (ARS)
2. Intrapersonal and interpersonal related stressors (IRS)
3. Teaching and learning-related stressors (TLRS)
4. Social related stressors (SRS)
5. Drive and desire related stressors (DRS)
6. Group activities related stressors (GARS)

The six groups are discussed further in the next chapter.

The validity of the MSSQ

The MSSQ consists of 40 items representing the six stressor domains. A validation study was conducted on 761 medical students representing multiple ethnicities, religions and cultures. The validation found that the MSSQ has good psychometric properties; it is a valid and reliable instrument that can be used to identify students' stressors as well as measure the intensity of the stressors. Factor analysis shows that all the items are well distributed according to the six groups. Reliability analysis shows that the MSSQ has a high internal consistency as Cronbach's alpha coefficient value was 0.95 which is more than the acceptable cut-off point of 0.6 (35). The Cronbach's alpha coefficient values for each domain are shown in table 1 below:

Table 1: The Cronbach's alpha value for each stressor domain.

Stressor Domain	Cronbach's alpha value
Academic related stressors (ARS)	0.921
Intrapersonal and interpersonal related stressors (IRS)	0.895
Teaching and learning-related stressors (TLRS)	0.858
Social related stressors (SRS)	0.710
Drive and desire related stressors (DRS)	0.646
Group activities related stressors (GARS)	0.728

Significance of MSSQ scores

Having a high score in a particular stressor group generally indicates that you perceive events, conditions or situations from that particular group as causing you stress. The scores, however, do require your frank and honest response in order for it to be of any use. The scores are also affected by factors which can falsely increase or lower the scores, but generally the validity and reliability studies have indicated that the scores from the questionnaire are highly trustworthy.

Having an insight about our stressors can help us to understand the sources of our stress. On top of that, it helps us to improve our ways of handling stressors so that we can manage our stress better.

Mild, Moderate, High & Severe scores: what do they mean?

<table>
<tr><th>Stressor domain</th><th>Mild*</th><th>Moderate*</th><th>High*</th><th>Severe*</th></tr>
<tr><td>Academic related stressors (ARS)</td><td rowspan="6">Indicates that it does not cause any stress on you. Even if it does, it just causes mild stress.</td><td rowspan="6">Indicates that it reasonably causes stress on you. However you can manage it well.</td><td rowspan="6">Indicates that it causes a lot of stress on you. Your emotions seem to be disturbed by it. Your daily activities are mildly compromised due to it.</td><td rowspan="6">Indicates that it severely causes stress on you. It disturbs your emotions badly. Your daily activities are compromised due to it.</td></tr>
<tr><td>Intrapersonal and interpersonal related stressors (IRS)</td></tr>
<tr><td>Teaching and learning-related stressors (TLRS)</td></tr>
<tr><td>Social related stressors (SRS)</td></tr>
<tr><td>Drive and desire related stressors (DRS)</td></tr>
<tr><td>Group activities related stressors (GARS)</td></tr>
</table>

* Mean domain score:
0.00 – 1.00 = MILD, 1.01 – 2.00 = MODERATE, 2.01 – 3.00 = HIGH & 3.01 – 4.00 = SEVERE

Detailed Description of the Six Stressor Domains

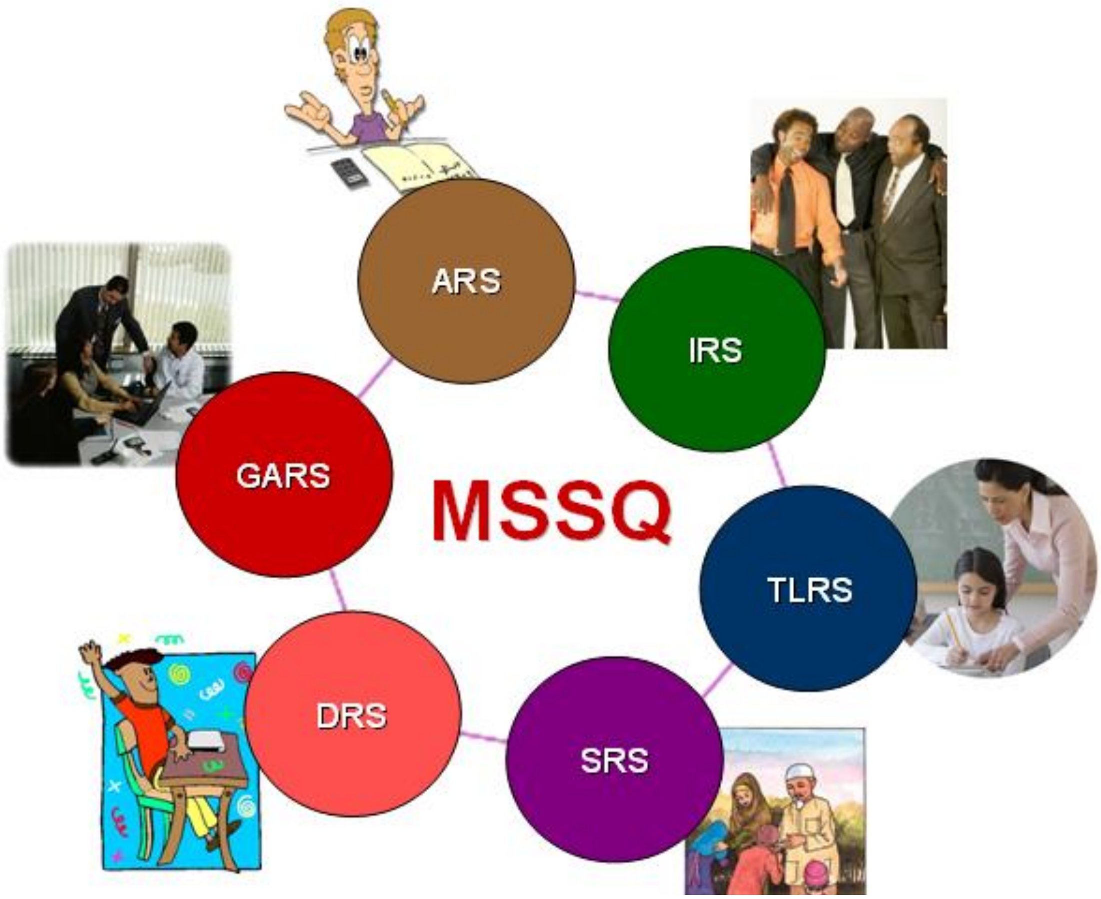

DOMAIN I: Academic Related Stressor (ARS)

Academic related stressors refer to any scholastic, university, college, educational or student events that cause stress on students. These include examination systems, assessment methods, grading methods, academic schedule, student activities related to academic events such as getting poor marks in examinations, high-self expectation to do well in studies, large amount of content to be studied, having

difficulty to understand content, lack of time to do revision, learning context full of competition, and having difficulty to answer questions given by teachers.

A high score in this domain indicates that academic matters are the main sources of stress. According to a study done on first year medical students from four different medical schools, those who perceived academic related stressors as causing high stress have 7 times higher risk to develop distress compared to those who perceived it as causing mild to moderate stress. Those who perceive it as causing severe stress have 16 times higher risk to develop distress compare to who perceive as causing mild to moderate stress. Many studies have reported that the major stressors of medical students were academic related (33, 34).

DOMAIN II: Interpersonal & Intrapersonal Related Stressor (IRS)

Interpersonal and intrapersonal related stressors refer to any form of relationships between and within individuals that cause stress. Intrapersonal stressors generally relate to relationships within one's own self, including poor motivation to study and self-conflict. Interpersonal stressors generally relate to relationships between individuals including as verbal, physical and emotional abuse caused by other persons, and conflict with personnel, teachers, colleagues, and staff.

A high score in this domain indicates that intrapersonal and interpersonal relationships are the main sources of stress.

DOMAIN III: Teaching and Learning Related Stressor (TLRS)

Teaching and learning related stressors refer to any events related to teaching or learning that causes stress. It is generally related to the appropriateness of tasks given by teachers to students, teachers' competency to supervise and teach students, quality of feedback given by teachers to students, recognition and support given by teachers to students, and clarity of learning objectives given by teachers to students.

A high score in this domain indicates that teaching and learning events are the main sources of stress. Consequently, it indicates that teaching and learning activities in the institution are unfriendly to students. This requires looking at components of teaching and learning process to determine the causes of stress on the students.

DOMAIN IV: Social Related Stressor (SRS)

Social related stressors refer to any form of community and societal relationships that cause stress. It generally relates to leisure time with family and friend, working with the public, private time for own self, working interruption by others, and facing patients' problems.

A high score in this domain indicates that societal and community events are the main sources of stress. This indirectly indicates that students have difficulty spending their time in social and community activities.

DOMAIN V: Drive & Desire Related Stressor (DRS)

Drive and desire related stressors refer to any form of internal or external forces that influence one's attitude, emotion, thought and behavior which subsequently cause stress. It generally relates to unwillingness to study medicine due to various reasons such as not being one's choice to study it, wrongly choosing the course, being demotivated after knowing the reality of medicine, parental wish to study medicine, and following friends to study medicine.

A high score in this domain indicates that drive and desire were the main sources of stress. According to the multicenter study done mentioned previously, those who perceived it as causing high stress have 3 times higher risk to develop distress compared to those who perceived it as causing mild to moderate stress. Those who perceive it as causing severe stress have 9 times higher risk to develop distress compared to who perceived as causing mild to moderate stress. It is understandable

because without drive and desire, one tend to give up and easily feel dissatisfied with everything which eventually leads to distress.

DOMAIN VI: Group Activities Related Stressor (GARS)

Group activities related stressors refer to any group events and interactions that cause stress. It generally relates to participation in group discussions, group presentations and others expectations to do well.

A high score in this domain indicates that group events and interactions are the main sources of stress. According to the multicenter study mentioned above, those who perceived it as causing moderate stress have 2 times higher risk to develop distress compared to those who perceived it as causing mild to moderate stress. Those who perceived it as causing high to severe stress have 4 times higher risk to develop distress compares to who perceived as causing mild to moderate stress. It is understandable as most of the educational activities in medicine involve group activities. Therefore, if someone is having difficulty with group activities then the person is easily distressed.

The Medical Student Stressor Questionnaire (MSSQ)

	Items
1	Tests/examinations
2	Talking to patients about personal problems*
3	Conflicts with other students
4	Quota system in examinations
5	Verbal or physical abuse by other student(s)
6	Parental wish for you to study medicine
7	Need to do well (self-expectation)
8	Not enough study material
9	Conflict with personnel(s)
10	Heavy workload
11	Participation in class discussion
12	Falling behind in reading schedule
13	Participation in class presentation
14	Lack of guidance from teacher (s)
15	Feeling of incompetence
16	Uncertainty of what is expected of me
17	Not enough medical skill practice*
18	Lack of time for family and friends
19	Learning context – full of competition
20	Teacher – lack of teaching skills

**** If your are not in clinical year yet, please answer based on what do you feel if you face this situation.***

Glossary:

Verbal Abuse is defined as to speak insultingly, harshly and unjustly about a person.
Physical Abuse is defined as to treat in harmful, injurious or offensive way to a person.

	Items
21	Unable to answer questions from patients*
22	Inappropriate assignments
23	Having difficulty understanding the content
24	Facing illness or death of the patients*
25	Getting poor marks
26	Poor motivation to learn
27	Lack of time to review what have been learnt
28	Verbal or physical abuse by teacher(s)
29	Frequent interruption of my work by others
30	Unable to answer the questions from the teachers
31	Conflict with teacher(s)
32	Unwillingness to study medicine
33	Large amount of content to be learnt
34	Need to do well (imposed by others)
35	Not enough feedback from teacher (s)
36	Unjustified grading process
37	Lack of recognition for work done
38	Working with computers
39	Verbal or physical abuse by personnel(s)
40	Family responsibilities

**** If your are not in clinical year yet, please answer based on what do you feel if you face this situation.***

Glossary:

Verbal Abuse is defined as to speak insultingly, harshly and unjustly about a person.
Physical Abuse is defined as to treat in harmful, injurious or offensive way to a person.

How to score the questionnaire

Below is the rating scale for each statement of MSSQ.

Rating Scales

0	1	2	3	4
Causing no stres at all	Causing mild stress	Causing moderate stress	Causing high stress	Causing severe stress

Below is the MSSQ Form to be filled by respondents based on the rating scales.

Section A	I	II	III	IV	V	VI
1						
2						
3						
4						
5						
6						
7						
8						
9						
10						
11						
12						
13						
14						
15						
16						
17						
18						
19						
20						
Total A						

Section B	I	II	III	IV	V	VI
21						
22						
23						
24						
25						
26						
27						
28						
29						
30						
31						
32						
33						
34						
35						
36						
37						
38						
39						
40						
Total B						

Below is the scoring method of MSSQ:

1st	I	II	III	IV	V	VI
Total A						
Total B						
Total						

2nd	I	II	III	IV	V	VI
Total						
Divided by	13	7	7	6	3	4
Score						

0 – 1.00	= CAUSE MILD STRESS
1.01 – 2.00	= CAUSE MODERATE STRESS
2.01 – 3.00	= CAUSE HIGH STRESS
3.01 – 4.00	= CAUSE SEVERE STRESS

Where to get the full version of MSSQ

The MSSQ can be obtained for free. To obtain full version of MSSQ and MSSQ Form please contact Dr Muhamad Saiful Bahri Yusoff through email: msaiful@kb.usm.my.

Disclaimers:

1) *Researchers are allowed to use this inventory for non-profit purposes and are permitted to duplicate or photocopy the MSSQ and MSSQ form as much as needed as long as proper citation (as shown below) and acknowledgement are given.*

   Muhamad S B Yusoff, Ahmad F A Rahim and Mohd J Yaacob. The development and validity of the Medical Student Stressor Questionnaire (MSSQ), *ASEAN Journal of Psychiatry*, Jan-June 2010; 11 (1). Available online at http://www.aseanjournalofpsychiatry.org/oe11105.htm

2) *Upon completion of your study, raw data of the MSSQ must be submitted to Dr Muhamad Saiful Bahri Yusoff through email msaiful@kb.usm.my. The raw data will only be used for below reasons:*
   a. *To establish population distribution reference score according to age, gender and ethnic groups.*
   b. *To establish the validity and reliability of MSSQ across samples.*

References

1. Rosenham, D. L., & Seligman, M. E. *Abnormal psychology (2nd ed.)*. New York: Norton, 1989.

2. Selye, H. *Stress without distress*. New York: Harper & Row, 1974.

3. Myers D.G. Stress and Health, in: *Exploring Psychology* (6th edn), pp. 402. New York: Worth Publishers, 2005.

4. Lazarus R.S. Theory-Based Stress Measurement, *Psychology Inquiry*, 1990: **1** (1); 3-13.

5. Lazarus, R. S., & Folkman, S. *Stress, appraisal, and coping.* New York: Springer, 1984.

6. Linn, B. S. & Zeppa, R. Stress in junior medical students: relationship to personality and performance. *J Med Educ,* 1984: **59**(1); 7-12.

7. Gugliemi R.S. & Tatrow K. Occupational stress, burnout, and health in teachers: a methodology and theoretical, *Review of Educational Research*, 1998: 68 (1); 61-69.

8. Karasek R.A & Theorell T. *Healthy Work*. New York: Basic Books, 1990.

9. Van Harrison R. Person-environment fit and job stress in: C.L. COOPER & R. PAYNE (Eds) Stress at Work. Chichester, UK: Wiley; 1978.

10. Filley A.C. & House R.J. *Managerial Process and Organizational Behaviour*, Scott Foresman, 1969.

11. Siegrist J. Adverse health effects of high-effort/low-reward conditions, *Journal of Occupational Health Psychology*, 1996: 1; 27-41.

12. Brissie J.S., Hoover-Demprey K.V. & Bassler O.C. Individual, situational contributors to teacher burnout, *Journal of Educational Research*, 1988: 82 (2); 106-112.

13. Muhamad S.B.Y, Ahmad F.A.R and Yaacob MJ. *Prevalence and sources of stress among medical students in Universiti Sains Malaysia* [dissertation]. Medical Education: Universiti Sains Malaysia (USM), Mei 2009.

14. Muhamad S.B.Y, Ahmad F.A.R and Yaacob M.J. The development and validity of the Medical Student Stressor Questionnaire (MSSQ), *ASEAN Journal of Psychiatry*, Jan-June 2010; 11 (1). Available online: http://www.aseanjournalofpsychiatry.org/oe11105.htm

15. Zaid, Z. A., Chan, S. C. & Ho, J. J. (2007). Emotional disorders among medical students in a Malaysian private medical school. *Singapore Med J,* **48**(10)**,** 895-899

16. Sherina MS, Lekhraj R, Nadarajan K (2003). Prevalence of emotional disorder among medical students in a Malaysian university, *Asia Pacific Family Medicine*, **2**, 213-217.

17. Saipanish, R. Stress among medical students in a Thai medical school. *Med Teach,* 2003: **25**(5)**;** 502-506.

18. Miller, P. M. & Surtees, P. G. (1991). Psychological symptoms and their course in first-year medical students as assessed by the Interval General Health Questionnaire (I-GHQ). *Br J Psychiatry,* **159,** 199-207.

19. Payne R.L & Fletcher B. Job demands, supports and constraints as predictors of psychological strain among school teachers, *Journal of Vocational Behaviour*, 1983: 22; 136-137.

20. Ko SM, Kua EH, Fones CSL (1999). Stress and the undergraduate, *Singapore Med. J*, **40**: 627-630.

21. Aktekin M., Karaman T., Senol Y.Y., Erdem S., Erengin H. & Akaydin M. Anxiety, depression and stressful life events among medical students: a prospective study in Antalya, Turkey. Medical Education, 2001; 35(1): 12-17.

22. Silver, H. K. & Glicken, A. D. (1990). Medical student abuse. Incidence, severity, and significance. *Jama,* **263**(4)**,** 527-532.

23. Rosal, M. C., Ockene, I. S., Ockene, J. K., Barrett, S. V., Ma, Y. & Hebert, J. R. A longitudinal study of students' depression at one medical school. *Acad Med,* 1997: **72**(6)**;** 542-546.

24. Shapiro, S. L., Shapiro, D. E. & Schwartz, G. E. Stress management in medical education: a review of the literature. *Acad Med,* 2000: **75**(7)**;** 748-759.

25. Clark, E. J. & Rieker, P. P. Gender differences in relationships and stress of medical and law students. *J Med Educ,* 1986: **61**(1)**;** 32-40.

26. Niemi, P. M. & Vainiomaki, P. T. (2006). Medical students' distress - quality, continuity and gender differences during a six-year medical programme. *Med Teach,* **28**(2)**,** 136-141.

27. Niemi, P. M. & Vainiomaki, P. T. (1999). Medical students' academic distress, coping and achievement strategies during the pre-clinical years, *Teaching & Learning in Medicine,* **11**(3)**,** 125-134.

28. Pickard, M., Bates, L., Dorian, M., Greig, H. & Saint, D. (2000). Alcohol and drug use in second-year medical students at the University of Leeds. *Med Educ,* **34**(2)**,** 148-150.

29. Newbury-Birch, D., White, M. & Kamali, F. (2000). Factors influencing alcohol and illicit drug use amongst medical students. *Drug Alcohol Depend,* **59**(2)**,** 125-130.

30. Flaherty, J. A. & Richman, J. A. (1993). Substance use and addiction among medical students, residents, and physicians. *Psychiatric Clin North Am,* **16**(1)**,** 189-197.

31. Liselotte N. Dyrbye, Matthew R. Thomas and Tait D. Shanafelt. Medical students distress: causes, consequences, and proposed solutions, *Mayo Clin Proc*, 2005: **80** (12); 1613-1622.

32. Guthrie E.A., Black D., Shaw C.M., Hamilton J., Creed F.H. & Tomenson B. Embarking upon a medical career: psychological morbidity in first year medical students. *Med Educ*, 1995; 29(5): 337-341.

33. Kaufman D.M, Day V. & Mensink D. Stressors in 1st-year medical school: comparison of a conventional and problem-based curriculum, *Teaching and Learning in Medicine*, 1996; 8(4), 188-194.

34. Kaufman D.M, Day V. & Mensink D. Stressors in Medical School: Relation to curriculum format and year of study, *Teaching and Learning in Medicine*, 1998; 10(3), 188-194.

35. Downing S.M. Reliability: on the reproducibility of assessment data, Medical Education, 2004; 38: 1006-1012.

36. Hays, L. R., Cheever, T. & Patel, P. Medical student suicide, 1989-1994. *Am J Psychiatry,* 1996; **153**(4)**:** 553-555.

ISBN 978-967-5547-01-0

9 789675 547010

View publication stats
